# Copper oxalate formation by lichens and fungi

**DOI:** 10.1038/s41598-021-03600-5

**Published:** 2021-12-20

**Authors:** Olga V. Frank-Kamenetskaya, Marina S. Zelenskaya, Alina R. Izatulina, Oleg S. Vereshchagin, Dmitry Yu. Vlasov, Dmitry E. Himelbrant, Dmitrii V. Pankin

**Affiliations:** 1 Institute of Earth Sciences, St. Petersburg State University, University emb., 7/9, St. Petersburg, Russian Federation 199034; 2 Faculty of Biology, St. Petersburg State University, University emb., 7/9, St. Petersburg, Russian Federation 199034; 3 Komarov Botanical Institute of the Russian Academy of Sciences, Professora Popova str., 2, St. Petersburg, Russian Federation 1973762; 4 Center for Optical and Laser Materials Research, St. Petersburg State University, Ulianovskaya str., 5, Peterhof, St. Petersburg, Russian Federation 198504

**Keywords:** Environmental microbiology, Mineralogy, Environmental chemistry

## Abstract

The present work focuses on the revealing the patterns of copper oxalates formation under the influence of lichens and fungi by combination of the results of field studies and model experiments. These findings create the scientific basis for the potential microbial technology applications (ore enrichment, monuments conservation, environment bioremediation, etc.). Copper oxalate moolooite Cu(C_2_O_4_)·H_2_O was discovered in saxicolous lichen *Lecidea inops* on the weathered chalcopyrite ore of Voronov Bor deposit (Central Karelia, Russia). Bioinspired syntheses of moolooite and wheatleyite Na_2_Cu(C_2_O_4_)_2_ 2H_2_O with the participation of the microscopic fungi *Aspergillus niger* (active producer of oxalic acid) were carried out on weathered Cu-ore from the Voronov Bor deposit. It was shown that morphology of moolooite crystals is controlled both by the underlying rock and by the species composition of microorganisms. Iron ions (sourced from the underlying rock) in the crystallization medium inhibits the moolooite formation. The observed intensive dissolution of moolooite crystals are well explained by washing effect of the intratalline solutions which depends on repeatedly dehydration / rehydration cycles in the lichens. Joint interpretation of original and published data shows that moolooite along with other cooper oxalates are biominerals.

## Introduction

Lithobiotic microbial community (lichens, microscopic fungi, bacteria) play an important role in rock weathering which often results in formation of insoluble oxalic acid salts (oxalates)^1,2^ on a rock surface. Investigation of these biominerals contributes to the essential questions on mechanisms of microbial oxalate biomineralization as well as modern mineral formation at nano- and micro- levels. Significant interest of the scientific community in moolooite and other copper oxalates is associated, first of all, with the development of biotechnologies for extracting valuable metals from ores, as well as with methods and approaches for neutralizing various media from toxic metals using microscopic fungi (micromycetes)^3–5^. Unique properties of copper compounds are widely used in modern nanotechnologies^1,6^. For example, copper oxalate has unusual antiferromagnetic properties and also serves as a precursor for the production of widely used nanoparticles CuO, Cu, Cu(OH)_2_ and Cu_2_O^7,8^. Copper oxide (analog of the tenorite mineral), for instance, has p-type semiconductivity^9^ and has potential applications in areas such as gas sensors, solar energy and catalysis^6,10^.

Copper oxalates (e.g., moolooite, Table S1) are the second most common oxalates in nature after calcium oxalates (whewellite and weddellite). Moolooite, Cu(C_2_O_4_)·H_2_O, is the most widespread naturally occurring copper oxalate. This biomineral was discovered in 1986 in bird guano deposit on chalcopyrite-bearing quartz outcrops in Mooloo Downs Station, Western Australia^11^. It is worth noting, that natural Cu(C_2_O_4_)·H_2_O was found in lichens earlier (Table S1), but was not described as a new mineral specie. Usually moolooite is found in biofilms with a predominance of lichens in association with various copper minerals.

Lichens, which are known as a moolooite accumulators (Table S1), are typically crustose saxicolous species and normally grow on natural siliceous rocks in well-lit habitats in mountains and flatlands^12–14^. Only rarely *Lecanora polytropa* (Ehrh. ex Hoffm.) Rabenh. can be found as lignicolous or on various anthropogenic substrates. Although cosmopolitan lichens *L. polytropa* and *Lecidea lactea* Flörke ex Schaer. can inhabit a wide range of siliceous substrates. Northern Hemispherical species *Acarospora rugulosa* Körb. and Eurasian *Lecidea inops* Th. Fr. prefer copper- and iron-bearing, or copper-bearing rocks, respectively. According to the data of numerous experiments (Table S2), copper oxalates confined to biofilms can also be formed under the action of fungi from different systematic and ecological groups.

Most of the copper oxalates (middlebackite, Cu_2_(C_2_O_4_)(OH)_2_; fiemmeite Cu_2_(C_2_O_4_)(OH)_2_ 2H_2_O; wheatleyite Na_2_Cu(C_2_O_4_)_2_ 2H_2_O and antipinite KNa_3_Cu_2_(C_2_O_4_)_4_) are rarely found in nature (Table S1). Some of them (e.g., wheatleyite and antipinite) were formed as a result of the interaction of metabolic products of living organisms (animals, birds) with associated copper minerals. It is assumed that the source of oxalic acid was coalified wood with plant remains in the case of fiemmeite. The source of oxalic acid for middlebackite formation is unknown.

The objective of this work is to reveal the patterns of copper oxalates formation under the influence of lichens and fungi, by jointly interpreting the results of field studies and model experiments. In particular, we assumed: (1) to study morphology of moolooite in the lichen thalli on the weathered chalcopyrite ore, as well to identify associated minerals and species of microorganisms (lichens and fungi); (2) to research in vitro the morphogenetic patterns of the formation of copper oxalates on the chalcopyrite ore by fungus *Aspergillus niger* (which is an active producer of oxalic acid); (3) to reveal the components of lithobiotic systems controlling the morphogenetic patterns of copper oxalates formation and discuss a possible mechanisms of their influence.

## Materials and methods

### Gelogical settings and sample materials

The Voronov Bor deposit was discovered in 1771 and was mined for a short time in the late 19th and early twentieth centuries. The ore-bearing strata are represented by quartzite-sandstones, quartz gravelstones, metabasalts and gabbro-diabases. This is a small deposit of sulfide copper ores, belonging to the type of "cuprous sandstones"^15^.

The Voronov Bor ore body has the shape of a lens 300 m long, 3–12 m thick, traced to 120–150 m^15,16^. The ores are disseminated and nested-veinlet with a sulfide content of 5–40%. The veins are more often subvertical, short; the nests have the appearance of “porphyry” segregations. The deposit contains an average of 1.3% Cu, with the maximum contents reaching up to 6% Cu^16^. The deposit shows ore zoning, which manifested itself in the change of chalcopyrite mineralization bornite-chalcopyrite and oxidized bornite-chalcocite. Respectively, according to the mineral composition, one can distinguish essentially chalcopyrite (CuFeS_2_) and bornite (Cu_5_FeS_4_) ores^17^.

**Figure 1:**
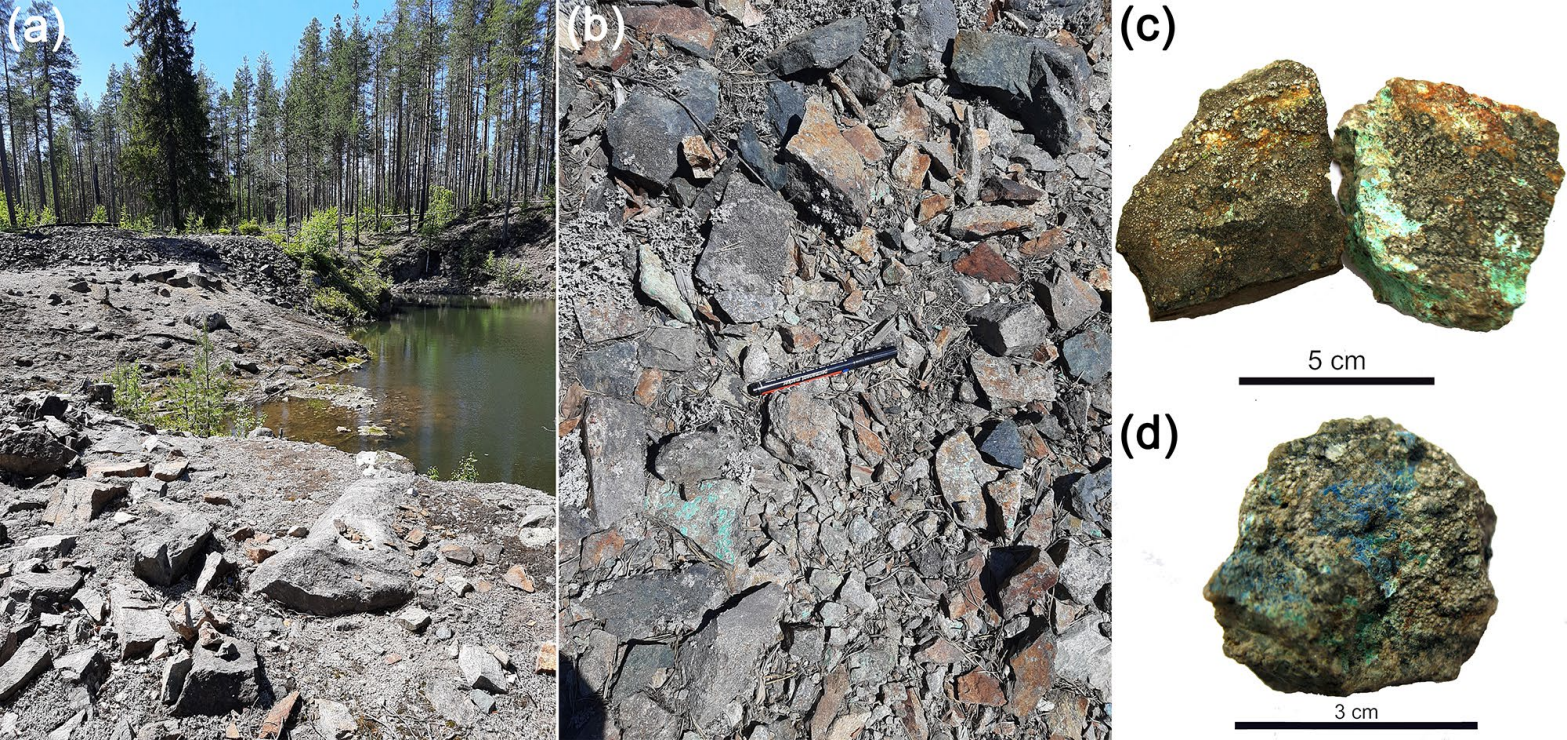
Copper ore samples from dumps of the Voronov Bor deposit (Central Karelia, Russia): (**a**) general view of dump, (**b**) Cu-ore fragments and vast rock, (**c**) malachite (green) and chalcopyrite (yellow), (**d**) azurite (blue).

Twenty-five fragments of weathered sulfide copper ore partially covered with crustose lichens biofilms were collected from the dumps of Voronov Bor deposit in July 2019 (Fig. 1).

### Model experiments

In order to reveal the contribution of microscopic fungi to the formation of cooper oxalates model experiments with the participation of the microscopic fungus *Aspergillus niger* (*A. niger*), which is an active producer of oxalic acid^18^, were carried out. This fungus is known for its ability to colonize a different natural and anthropogenic substrates in a variety environment. Due to its high metabolic activity, *A. niger* causes the transformation of mineral substrates and formation of secondary minerals that allows it to be actively used in modern biotechnologies^2^. Industrial sources of copper oxalate production using of *A. niger* are given in Table S2.

*Aspergillus niger* (strain Ch4/07) was isolated from the damaged surface of Proconesos marble (Chersonesos, Crimea). The species identification of the strain was based on the sequence of the ITS region of rDNA (GenBank accession no. KF768341). Experiments were carried out at room temperature (20–25 °C) in a liquid Czapek-Dox nutrient medium (g/l: NaNO_3_—2.0; KH_2_PO_4_—1.0; MgSO_4_·7H_2_O—0.5; KCl—0.5 FeSO_4_·7H_2_O—0.01, glucose—30.0 g/l). The initial pH of the medium was 5.5. Fragments of secondary Cu-bearing phases on the surface of chalcopyrite ore from Voronov Bor deposit (size ~ 5 × 5 mm) were placed on the bottom of a Petri dishes and 15 ml of liquid Czapek-Dox medium was added, so that their surface was completely covered. Inoculation was performed with conidia of a microscopic fungus of a 10-day culture grown on a solid nutrient medium of Czapek-Dox. The cultivation time was 5–6 days and the experiments were performed in triplicate with constant monitoring of the medium pH. The pH values during the experiment were evaluated using pH-meter Checker 1 (HI 98103).

### Methods

Optical microscopy was used to study petrographic features and mineral composition of copper ore and products of its weathering, as well as for identification of microorganisms. Thin sections of a copper ore were studied under a petrographic microscope Leica DLMP. Lichen thalli and microscopic fungi cultures samples were examined using a Leica MZ16 stereo microscope and Leica DM300 LED microscope. Identification of lichens was performed according to the standard procedure^14,19^. Modern taxonomy of lichens was given according to Santesson's Checklist of Fennoscandian Lichen-forming and Lichenicolous Fungi^11^ and Cumulative Checklist for the Lichen-Forming, Lichenicolous and Allied Fungi of the Continental United States and Canada^20^. Micromycetes from biofilms were identified after isolation to the nutrient media in accordance with guide books and monographs^21–24^. Modern taxonomy of microscopic fungi was given by Index Fungorum^25^.

Powder X-ray diffraction (PXRD) was used to determine the mineral composition of the underlying mineral substrate and to determine the phase composition of crystals in biofilms and products of model experiments. Measurements were performed using Rigaku "MiniFlex II" and Bruker "D2 Phaser" powder diffractometers (CuKα radiation, λ = 1.54178 Å). X-ray diffraction patterns were collected at room temperature in the range of 3°–80° 2θ with a step of 0.02°. Phase identification was carried out using the ICDD PDF-2 Database (release 2016).

Raman spectrum of moolooite was obtained at LabRam HR 800 (Horiba Jobin–Yvon) confocal Raman spectrometer with He–Ne laser excitation (632.8 nm). The laser power was about 1mW and focused by 100 × objective to a point approximately 2 µm^2^. Raman spectrum was obtained in backscattering geometry. The diameter of aperture was 150 μm, and the 600 gr/mm grating was used. The spectrum was obtained in the range of 40–4000 cm^–1^ with resolution of 2 cm^–1^ at room temperature. Accumulation time was 150 s with 2–4 repetitions. The spectra were processed using licensed ASD LabSpec 5.0 and Origin 2019 software. Moolooite identification was carried out using typical Raman bands (cm^−1^)^26^: 210, 300 (lattice modes), 558 (ν (M–O) + ν (C–C)), 585, 611 (water vibration), 831 (νs (C–O)/δ (O–C–O)), 923 (νs (C–O) + δ (O–C=O)), 1486 (νs (C–O) + ν (C–C)), 1515 (νa (C=O).

Morphological features and element composition of copper oxalate crystals found in lichen’s thalli and obtained during model experiments (unpolished samples) were studied using scanning electron microscopes (SEM) Zeiss Merlin, Tescan MIRA3 LMU and Hitachi TM3000, equipped with OXFORD energy dispersive microanalysis attachment.

The elemental composition of natural and synthesized oxalate crystals, as well as of Cu-ore substrate were studied on carbon-coated polished samples using Hitachi S-3400N SEM equipped with an Oxford Instruments AzTec Energy X-Max 20 energy dispersive (EDX) and INCA WAVE 500 wavelength-dispersive (WDX) spectrometers. Conditions of the experiment were: 20 kV accelerating voltage, 1 nA beam current, and 30 s data-collection time (excluding dead time). Synthetic material and minerals were used as standards.

## Results

### Underlying mineral substrate characterization

**Figure 2:**
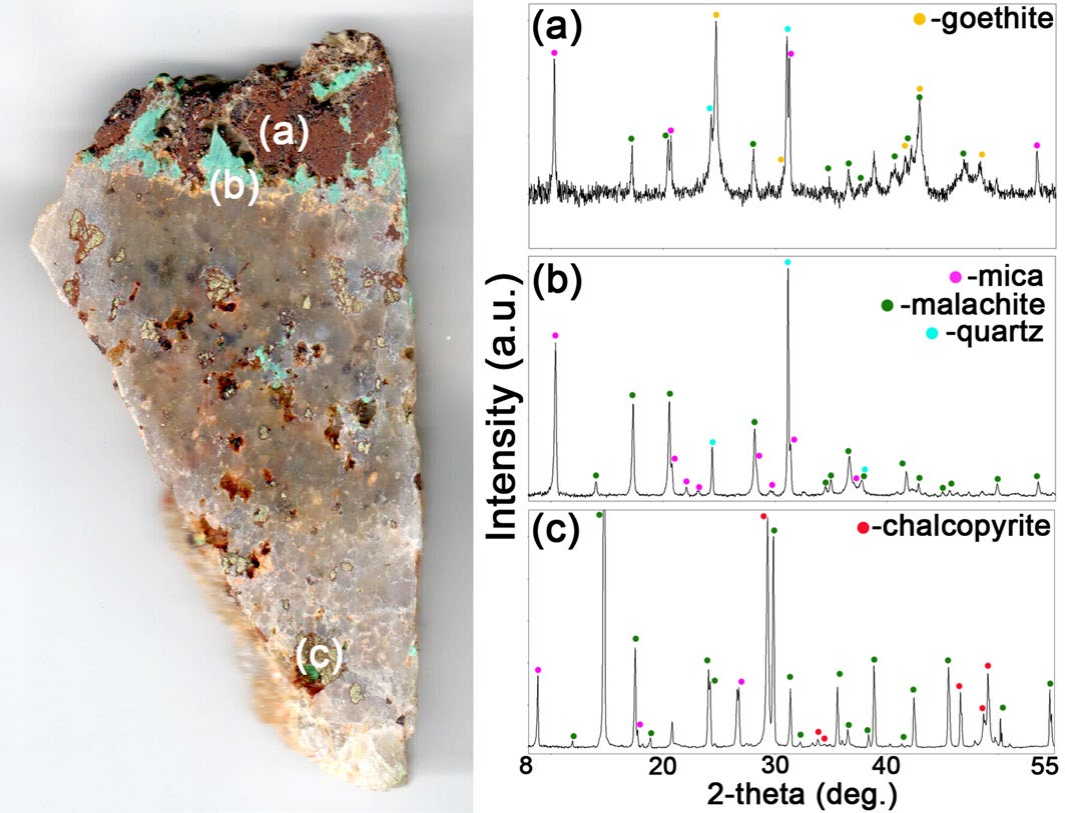
Powder X-ray diffraction patterns (Rigaku PDXL 2.0) of minerals from the Voronov Bor deposit: (**a**) goethite; (**b**) malachite, mica and quartz; (**c**) chalcopyrite, malachite and mica.

According to PXRD (Fig. 2) the main minerals of weathered Cu-ore samples from the Voronov Bor deposit are chalcopyrite, quartz, mica, goethite and malachite. All collected samples belong to chalcopyrite ore type^15^. Besides that, outer parts of the samples are enriched in X-ray amorphous phase.

**Figure 3:**
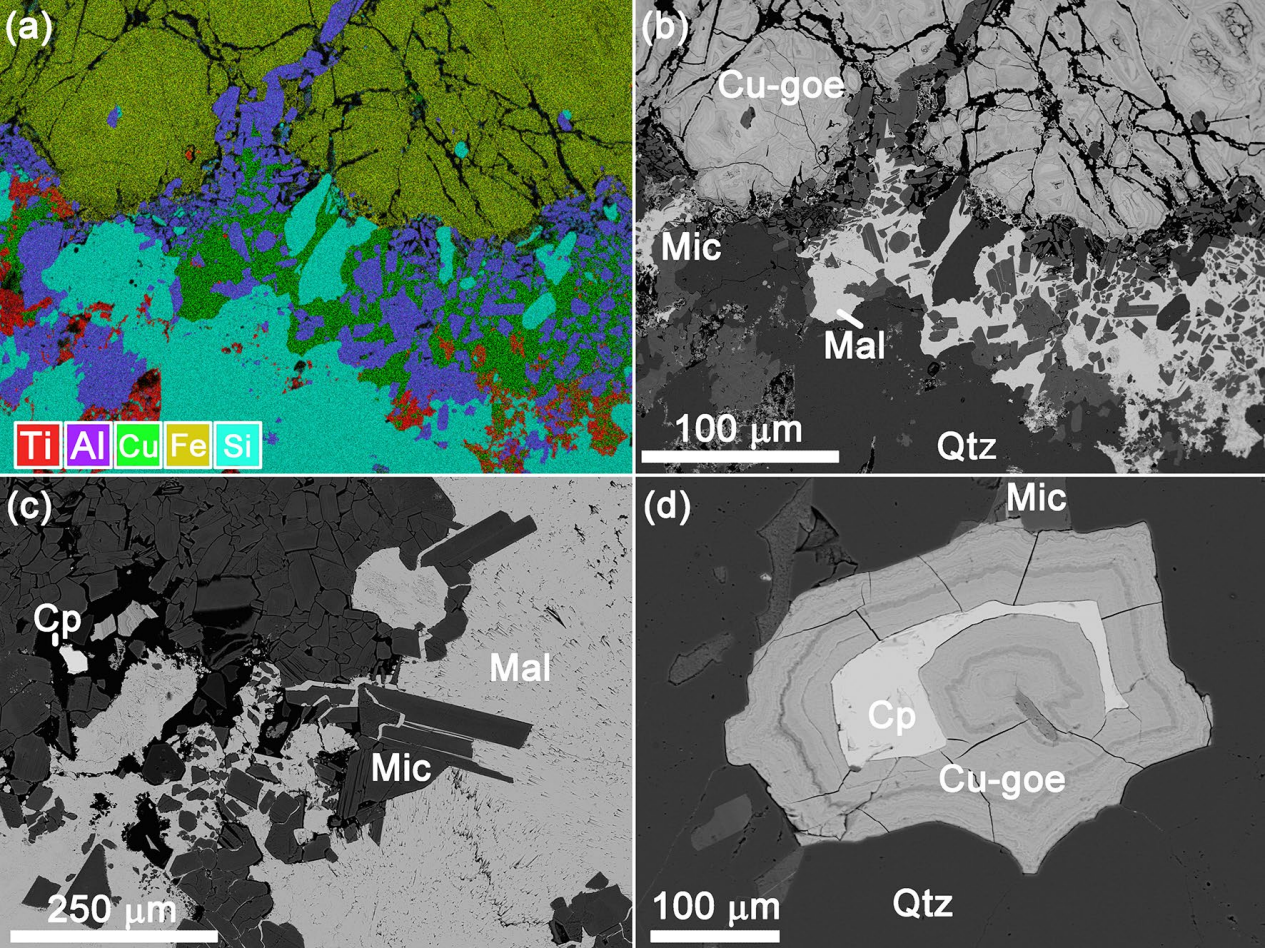
Weathered Cu-ore: (**a**) elemental map (AZtec SmartMap software, Oxford Instruments); (**b**) quartz vein with malachite; (**c**) isometric mica crystals with malachite; (**d**) chalcopyrite grain, covered with goethite. *Note* Cp, chalcopyrite; Cu-goe, goethite and Cu, Fe-rich phase; Mic, mica; Mal, malachite; Qtz, quartz.

According to SEM-EDX, quartz veins hosting isometric chalcopyrite grains up to 3 mm in size and elongated mica (aluminoceladonite?) crystals (enriched with copper up to ~ 2 wt% CuO) (Fig. 3). In all cases primary minerals are strongly weathered: chalcopyrite is covered with Cu-carbonates (malachite and azurite) and Cu-rich (up to ~ 15 wt% CuO) reddish ferruginous bloom, consisting of goethite and Cu, Fe-rich X-ray amorphous phase. Barite, TiO_2_ (rutile?) and iodargyrite were also found.

### Species composition of biofilms on weathered sulfide copper ore

**Figure 4:**
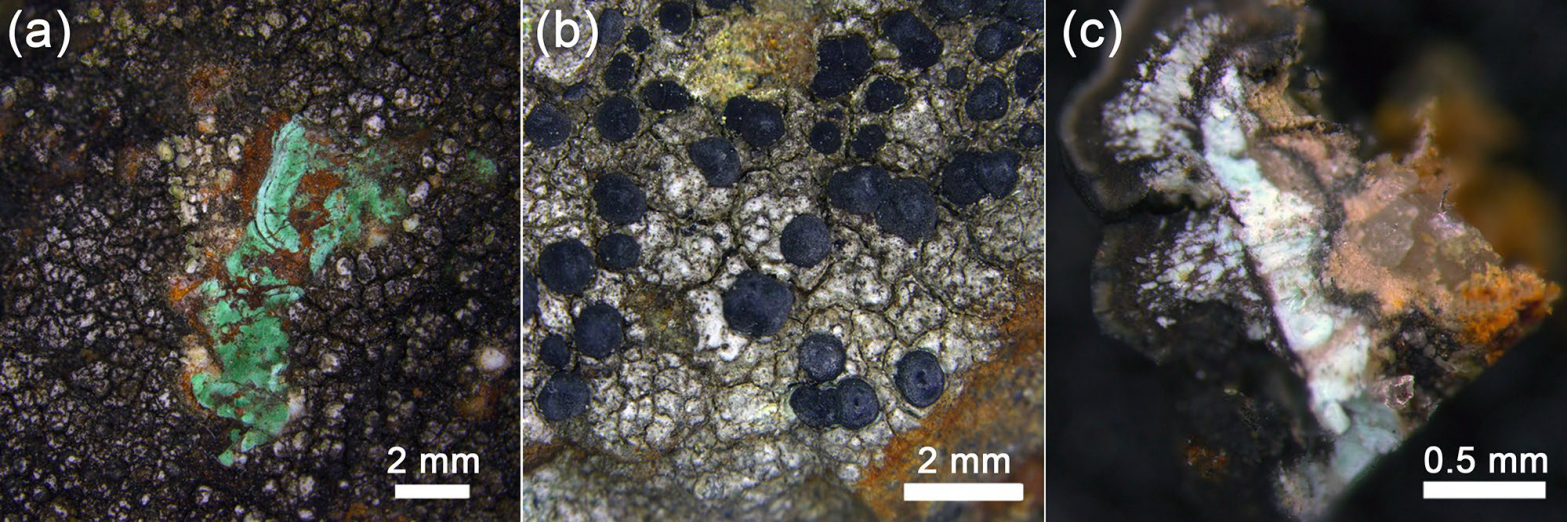
The lichen *L. inops* on the surface of sulfide Cu-ore of the Voronov Bor: (**a**) black lichen apothecia on green malachite, (**b**) black lichen apothecia on reddish ferruginous bloom and chalcopyrite, (**c**) bluish moolooite crystals in apothecia.

On the surface of the chalcopyrite ore from the Voronov Bor deposit, 5 species of crustose lichens and 18 species of micromycetes were found (Table S3). Among lichens (Fig. 4), there is saxicolous lichen *L. inops* (Fig. 4a, b), which, is well-known inhabitant of communities developing on Cu-bearing minerals (Table S1). Other detected crustose lichens are also known as saxicolous species growing mainly on siliceous, sometimes slightly basic substrates in well-lit habitats in mountains and plains^12–14^. *Rhizocarpon lavatum*is known as preferring damp siliceous rocks and stones. *R. lavatum* and *R. inarense*are widely distributed in North Hemisphere, whereas *Schaereria fuscocinerea* is a cosmopolitan lichen. All three species are not known as preferring copper-bearing minerals.

### Copper oxalate moolooite in lichen thalli on weathered copper sulfide ore

**Figure 5:**
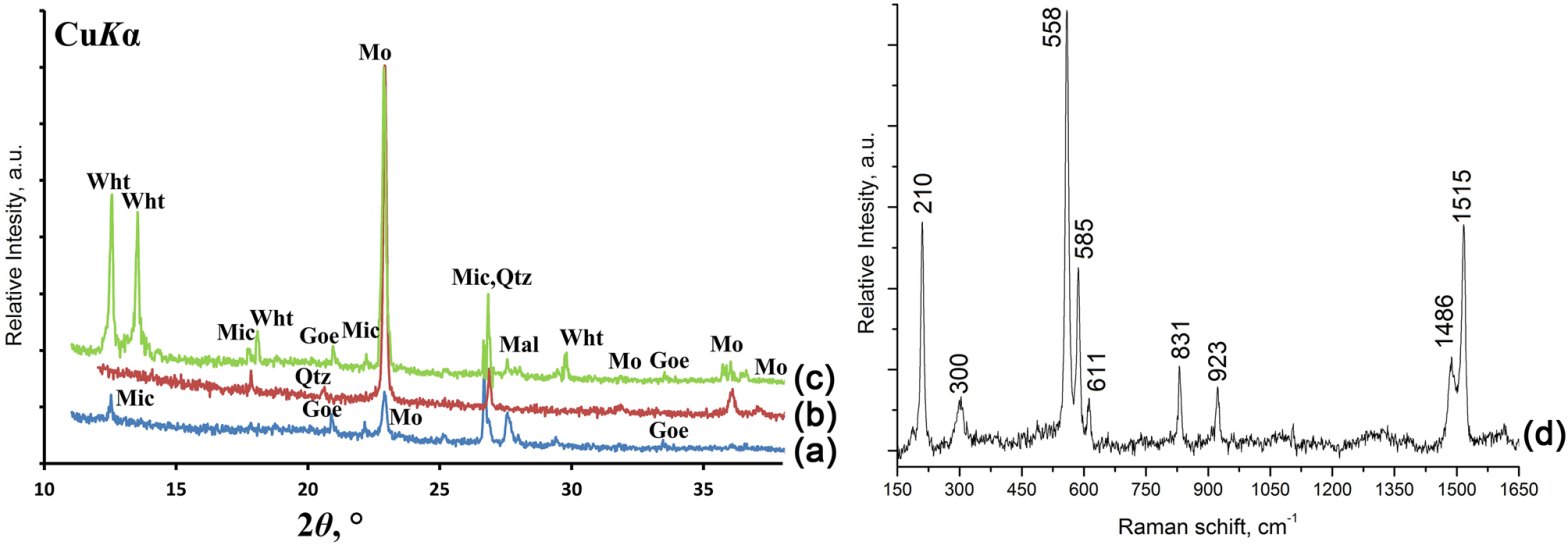
Copper oxalates identification: (**a**) PXRD pattern (Rigaku PDXL 2.0) of moolooite from *L. inops* on ore from the Voronov Bor deposit; (**b**) PXRD pattern (Rigaku PDXL 2.0) of moolooite formed in model experiment on malachite from the Voronov Bor deposit (6 days); (**c**) PXRD pattern (Rigaku PDXL 2.0) of moolooite and wheatleyite formed in model experiment on malachite from the Voronov Bor deposit (5 days); (**d**) Raman spectra (Origin 2019) of moolooite crystals from *L. inops* on ore from the Voronov Bor deposit. *Note* Mo, moolooite; Wht, wheatleyite; Goe, goethite; Mic, mica; Mal, malachite; Qtz, quartz.

According to PXRD and Raman spectroscopy data (Fig. 5) moolooite in the dumps of the Voronov Bor deposit is present on the weathered surface of copper sulfide ore in thalli of the only lichen *L. inops* (Fig. 5a, d) and can be localized both in thallus and in apothecia (Fig. 4c). Moolooite crystals are confined to secondary formations on the surface of copper ore: copper carbonates (malachite and azurite), as well as reddish ferruginous bloom containing goethite and an Cu,Fe-rich X-ray amorphous phase.

**Figure 6:**
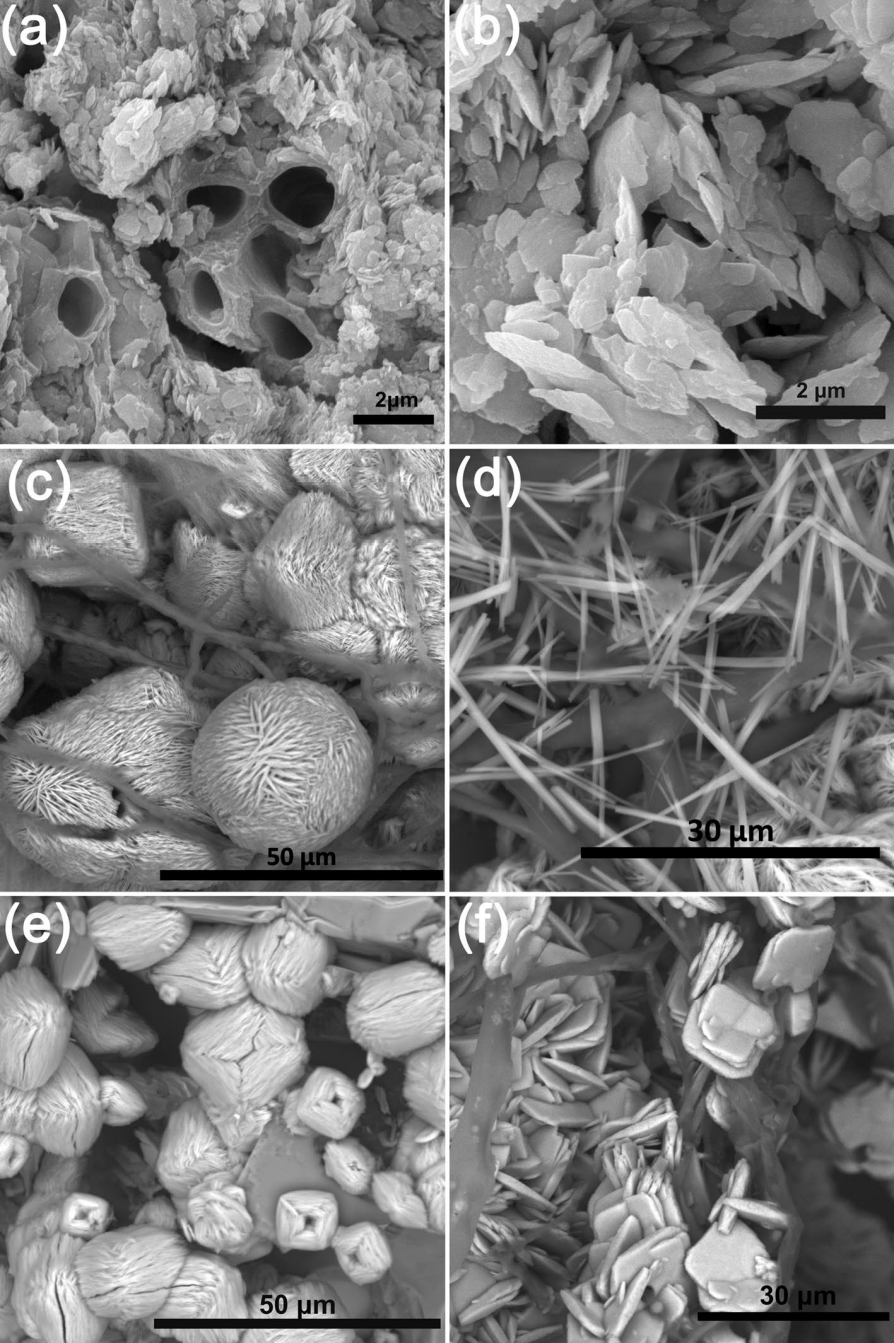
SEM images of cooper oxalate crystals: (**a**) mycobiont hyphae surrounded by moolooite crystals and their intergrowths (*L. inops*, Voronov Bor deposit); (**b**) aggregates of split moolooite crystals with rounded ribs (*L. inops*, Voronov Bor deposit), (**c**) spherulite-like intergrowths of moolooite crystals and acicular wheatleyite crystals on malachite (on the 5th day of the model experiment); (**d**) acicular wheatleyite crystals on malachite (on the 5th day of the model experiment); (**e**) spherulite-like intergrowths of moolooite crystals on azurite (on the 5th day of the model experiment); (**f**) the lamellar moolooite crystals and their intergrowths on a Cu, Fe-rich phase (on the 5th day of the model experiment).

SEM images clearly show that moolooite form small lamellar crystals ranging in size from 200 nm to 3 µm (Fig. 6), which are often confined to the hyphae of the lichen mycobiont (Fig. 6a). Almost all crystals are split, characterized by rounded edges, and often form intergrowths (Fig. 6b). According to EDX and WDX data, moolooite crystals found in the vegetative part of the lichen thallus may contain iron impurities (from 0.21 to 1.7 wt% FeO).

### Copper oxalates formed in vitro on the ore surface from the Voronov Bor under the action of the fungus *Aspergillus niger*

According to PXRD data moolooite in the form of numerous lamellar crystals and/or their intergrowths was recorded under experimental conditions with the participation of the microscopic fungus *A. niger* on the 5th day of the experiment in all secondary formations on the surface of copper ore from the Voronov Bor deposit: malachite (Fig. 5c), azurite, and reddish ferruginous bloom (goethite + Cu, Fe-rich X-ray amorphous phase). On the surface of malachite, water-soluble oxalate of Cu and Na, wheatleyite, was also detected (Fig. 5c), which then disappeared on the 6th day of the experiment (Fig. 5b).

On the surface of malachite and azurite, moolooite is represented by spherical intergrowths (sizes from 25 to 50 µm) (Fig. 6c, e) formed as the result of splitting of lamellar crystals. On the surface of the reddish ferruginous bloom the lamellar moolooite crystals are finer (10–15 μm) and form intergrowths in the form of stacks with a depression in the center (Fig. 6f), i.e. characterize an earlier stage of splitting (the beginning of the formation of a spherulite-like intergrowth). Wheatleyite is represented by acicular crystals (~ 0.5–2 µm thick), the length of which reaches 25 µm (Fig. 6c, d). According to EDX and WDX data, moolooite crystals, formed on a reddish ferruginous bloom enriched with Cu and Fe, contain Fe impurity in an amount ranges from 0.64 to 1.31 wt% FeO. No Fe impurities were detected in moolooite crystals formed on the surface of malachite and azurite.

## Discussion

Cooper oxalate moolooite have been discovered in the lichen thalli *L. inops* on the surface of copper ore in the dumps of the Voronov Bor deposit (Central Karelia, Russia) for the first time. We have described in detail the moolooite in biofilms from Karelia and have characterized the underlying and associated minerals and species of microorganisms (lichens and fungi), which accumulate mollooite.

The species diversity of lichens on the copper ore of the Voronov Bor deposit is rather small, which can be explained by the toxicity of divalent copper. Only those species that have passive physicochemical defense mechanisms selectively grow on such substrates, since they are able to bind copper in the form of intracellular organometallic complexes and in the form of extracellular copper oxalates^27–30^. Such lichens include the *L. inops* species, in which moolooite from Karelia was found. This and earlier findings of copper (Table S1) and calcium oxalates^31,32^ in *L. inops* lichen indicate that its mycobiont is capable of secreting oxalic acid. Among the micromycete species capable of producing oxalic acid, following were also detected on the copper ores: *P. brevicompactum, P. citrinum, P. decumbens, P. lanosum, P. oxalicum, P. waksmanii, P. pannorum, T. viride*^33–35^.

Morphology of moolooite crystals found in thalli of lichens on the surface of the Voronov Bor ore are closely related to moolooite crystals formed in biofilms on the surface of copper ores in other deposits (usually in association with various secondary copper minerals) (Table S1)^31,32,36^. In all the cases, moolooite is represented by small lamellar crystals (often formed intergrowths) with rounded edges, i.e. with traces of dissolution. It could be resulted from washing effect by the intratalline solutions due to repeatedly occur dehydration / rehydration cycles in the lichen thalli – a natural process depending from changes in weather (especially precipitation) and daily rhythms of humidity and temperature^37–39^. The presence of the organic acids, secreted by microscopic fungi in the intratalline solution, made this process more active.

Morphology of moolooite crystals found in biofilms that were formed on the surface of the copper ore from the Voronov Bor deposit, significantly differs from that for moolooite crystals obtained under the action of the fungus *A. niger* in laboratory conditions on the surface of the copper ore from the same deposit. Regular intergrowths of moolooite crystals (spherulite—and stacks-like) that were formed under laboratory conditions as a result of cleavage were not detected in biofilms. This feature is likely points to the fact that ionic supersaturation of the solutions and the moolooite crystal growth rate within biofilms are not as high as in the experimental conditions.

The results of bioinspired syntheses clearly demonstrated the effect of the elemental composition of the underlying substrate on oxalate crystallization. Comparison of the morphology of moolooite crystals formed on the surface of Cu- (malachite, azurite, Fig. 6c, e) and Cu–Fe substrates (goethite + Cu, Fe-rich X-ray amorphous phase; Fig. 6f) showed that an earlier stage of splitting is observed on the Fe-bearing substrate. Thus, iron cations inhibits an oxalate crystallization. It hasn’t yet been possible to separate the effect of pure Cu and Cu–Fe-bearing substrates on the morphology of moolooite crystals in biofilms. However, the fact that according to EDX and WDX data, Fe content in moolooite in biofilms and in bioinspired moolooite is very close (less than 2 wt% FeO) suggests that, Fe ions should also affect the crystallization of moolooite in the environment, either replacing (in very insignificant amounts) Cu cations, or being adsorbed on faces of growing moolooite crystals. To clarify these mechanisms, an additional research is needed.

Formation of water-soluble oxalate of Cu and Na, the wheatleyite, occurs due to the presence of oxalate ions in the medium (provided by the metabolism of fungi), Cu ions (sourced from the underlying substrate) and sodium ions (present in Czapek's nutrient medium). Formation of wheatleyite under the action of *A. niger* on the other Cu-bearing substrates (cuprite and brochantite) was reported in the recent paper of our workgroup^2^.

Single findings of wheatleyite in nature (Table S1) are well explained by the fact that it is water-soluble and can be stored for a long time only under special conditions. Probably, this can also explain the rare occurrence of other copper oxalates (middlebackite, fiemmeite, antipinite), but there still no data on their solubility. In addition, rare occurrence of wheatleyite and antipinite may be associated with the atypical associations of elements (Cu + Na and Cu + Na + K) for rocks and minerals, which should occur simultaneously in the environment during the formation of these minerals.

## Conclusion

On the base of jointly interpretation of the results of field studies and bioinspired syntheses the patterns of copper oxalates formation under the influence of lichens and fungi were revealed. We have found the effect of underlying rock, and microorganisms species composition on the morphology of moolooite. The high likelihood of moolooite finding in saxicolous lichen *L. inops* (well-known inhabitant of communities developing on copper-bearing minerals) was confirmed. Bioinspired syntheses of moolooite and wheatleyite with the participation of fungi have been successfully carried out.

It was shown that iron ions inhibits moolooite formation and, accordingly, affects the morphology of its crystals. The present of the intratalline solutions, the influence of which depends on repeatedly dehydration/rehydration cycles in the lynches, leads to an intensive dissolution of moolooite crystals in biofilms. At the level of hypothesis, an explanation for the rare occurrence of wheatleyite, middlebackite, fiemmeite, and antipinite in nature has been given. The results obtained by us as well as published data suggest that moolooite along with other cooper oxalates are biominerals. They are formed as products of the reaction of metabolic products of living organisms (lichens, fungi, birds, animals and other) with Cu-rich rocks and minerals.

## Supplementary Information

Supplementary Tables.
