# Supplementary material for: Copper oxalate formation by lichens and fungi
Source: Sci Rep. 2021 Dec 20;11:24239. doi: 10.1038/s41598-021-03600-5 (PMC8688476; doi:10.1038/s41598-021-03600-5)
Supplement: Supplementary file 1 — Supplementary Tables. [file 41598_2021_3600_MOESM1_ESM.docx]

**Supplementary material**

**Table S1.** Copper oxalates in nature

| **Sampling area** | **Oxalic acid source** | **Associated minerals -sources of copper** | **Reference** |
| --- | --- | --- | --- |
| Moolooite, Cu(C_2_O_4_)·*n*H_2_O | | | |
| Mine at Gjersvik, Nord Trøndelag, Norway,  Ramundberget, Sweden | Lichen (*Acarospora rugulosa, Lecidea lactea, Lecidea inops*) | Chalcopyrite, atacamite, brochantite | ^1^ |
| Coniston copper mines, the English Lake District, England | Lichen  (*L. inops*) | Malachite, azurite, chrysocolla, covelline, bornite | ^2^ |
| Mooloo Downs station  homestead, Western  Australia | Bird guano | Brochantite, antlerite, atacamite | ^3^ |
| Mine at Gjersvik, Nord Trøndelag, Norway,  Ramundberget, Sweden | Lichen (*A. rugulosa, L. lactea, L. inops*) | Chalcopyrite, atacamite, brochantite | ^4^ |
| Sarbai Deposit, Rudny, Kazakhstan | Bird guano | Sampleite, libethenite, brochantite, atacamite | ^5^ |
| Mine at Riddarhyttan Kopperverket, Bergslagen District, Sweden | Lichen *(Lecanora polytropa)* | Copper ore | ^6^ |
| Samuel site, Murmansk coast, the Kola region, Russia | not known | Chalcopyrite, brochantite | ^7^ |
| Middlebackite, Cu_2_(C_2_O_4_)(OH)_2_ | | | |
| Iron Monarch quarry, South Australia | not known | Atacamite, bornite, chalcocite, chalcopyrite, covellite, | ^8,9^ |
| Fiemmeite Cu_2_(C_2_O_4_)(OH)_2_·2H_2_O* | | | |
| Passo di San Lugano, Val di Fiemme, Trento, Italy | coalified woods / plant remains | Brochantite, cuprite, devilline, malachite, azurite, zeunerite/metazeunerite, tennantite, chalcocite | ^10^ |

| Wheatleyite Na_2_Cu(C_2_O_4_)_2_·2H_2_O | | | |
| --- | --- | --- | --- |
| Mine dumps of a Pb-Zn vein deposit. Wheatley mine, near Phoenixville, Pennsylvania, USA | animal waste | No data | ^11^ |
| Antipinite KNa_3_Cu_2_(C_2_O_4_)_4_ | | | |
| Pabellón de Pica Mountain, Iquique Province, Tarapacá Region, Chile | guano | Chanabayaite, joanneumite | ^12^ |

* Found in association with moolooite and middlebackite

**Table S2**. Copper oxalates formed *in vitro* under the action of fungi.

| ***Copper source*** | ***Fungi species*** | ***Reference*** |
| --- | --- | --- |
| **Moolooite Cu(C_2_O_4_)·*n*H_2_O** | | |
| Cuprite  Cu_2_O | *Aspergillus niger* | ^13^ |
| Copper sulfate  CuSO_4_·5H_2_O | *Aspergillus* spp. | ^14^ |
|  | *Poria placenta*  *Verticillium psalliotae*  *Aspergillus niger*  *Penicillium spinulosum*  *Penicillium* spp. | ^15^ |
| Brochantite (Cu_4_(SO_4_)(OH)_6_),  cuprite (Cu_2_O),  atacamite (Cu_2_(OH)_3_Cl)  Coins, copper roof sheets  Copper patinas | *Beauveria bassiana* | ^16–18^ |
| Copper phosphate  (Cu_3_(PO_4_)_2_·2H_2_O | *Beauveria caledonica* | ^19^ |
| Copper sheet pieces | *Aspergillus niger* | ^20^ |
| CuSO_4_ treated wood blocks | *Fomitopsis palustris*  *Antrodia xantha* | ^21^ |
| Malachite Cu_2_(CO_3_)(OH)_2_ | *Aspergillus niger* | ^22^ |
| Cuprite (Cu_2_O),  malachite (Cu_2_(CO_3_)(OH)_2_),  brochantite (Cu_4_(SO_4_)(OH)_6_) | *Aspergillus niger* | ^23^ |
| **Fiemmeite Cu_2_(C_2_O_4_)(OH)_2_·2H_2_O** | | |
| Malachite (Cu_2_(CO_3_)(OH)_2_) | *Aspergillus niger* | ^23^ |
| **WheatleyiteNa_2_Cu(C_2_O_4_)_2_·2H_2_O** | | |
| Brochantite  Cu_4_(SO_4_)(OH)_6_ | *Aspergillus niger* | ^23^ |

**Table S3.** The species composition of lichens and micromycetes in biofilms on Сu-ore from the Voronov Bor deposit.

| **Species of lichens** | **Species of micromycetes** |
| --- | --- |
| *Lecidea inops* Th. Fr.  *Rhizocarpon inarense* (Vain.) Vain.  *R.lavatum* (Fr.) Hazsl.  *Schaereria fuscocinerea* (Nyl.) Clauzade et Cl. Roux | *Arthrinium phaeospermum* (Corda) M.B. Ellis  *Aureobasidium pullulans* (de Bary & Löwenthal) G. Arnaud  *Cladosporium cladosporioides* (Fresen.) G.A. de Vries  *C.sphaerospermum* Penz.  *Coniosporium* sp.  *Fusarium oxysporum* Schltdl.  *Monodictys levis* (Wiltshire) S. Hughes  **Penicillium brevicompactum* Dierckx  **P.citrinum* Thom  **P.decumbens* Thom  **P.lanosum* Westling  **P.oxalicum* Currie & Thom  **P.waksmanii* K.W. Zaleski  **Pseudogymnoascus pannorum* (Link) Minnis & D.L. Lindner  *Scopulariopsis brumptii* Salv.-Duval  *Scytalidium lignicola* Pesante  *Sydowia polyspora* (Bref. & Tavel) E. Müll.  **Trichoderma viride* Pers. |

| *Micromycete species capable of producing organic acids including oxalic acid^24–26^ |
| --- |

Refernces:

1. Purvis, O. W. The Occurrence of Copper Oxalate in Lichens Growing on Copper Sulphide-Bearing Rocks In Scandinavia. *Lichenol.* **16**, 197–204 (1984).

2. Purvis, O. W. & James, P. W. Lichens of the coniston Copper mines. *Lichenol.* **17**, 221–237 (1985).

3. Clarke, R. M. & Williams, I. R. Moolooite, a naturally occurring hydrated copper oxalate from Western Australia. *Mineral. Mag.* **50**, 295–298 (1986).

4. Chisholm, J. E., Jones, G. C. & Purvis, O. W. Hydrated copper oxalate, moolooite, in lichens. *Mineral. Mag.* **51**, 715–718 (1987).

5. Chukanov, N. & Pekov, I. Moolooite, Cu(C2O4)•H2O, from the Sarbayskoye deposit – the first finding in the CIS. *Mater. Ural Mineral. Sch.* **Yekaterinb**, 183-185 (in Russian) (1996).

6. Purvis, O. W. *et al.* Mineral phases and element composition of the copper hyperaccumulator lichen Lecanora polytropa. *Mineral. Mag.* **72**, 607–616 (2008).

7. Voloshin, A., Karpov, S., Chernyavskiy, A. & Kompanchenko, A. New data on minerals. Issue 4. The first finds in Russia and in the Kola region. in *Proceedings of the Fersman Scientific Session of the Geological Institute KSC RAS* 95-102 (in Russian) (2018). doi:10.31241/FNS.2018.15.023.

8. Elliott, P. Middlebackite, a new Cu oxalate mineral from Iron Monarch, South Australia: Description and crystal structure. *Mineral. Mag.* **83**, 427–433 (2019).

9. Demartin, F., Campostrini, I., Ferretti, P. & Rocchetti, I. Secondo ritrovamento mondiale di middlebackite presso il Passo di San Lugano ( Carano , Trento , Italia ) Second global occurrence of middlebackite near the Passo di San Lugano ( Carano , Trento , Italy ). *Geo.Alp.* **14**, 35–38 (2017).

10. Demartin, F., Campostrini, I., Ferretti, P. & Rocchetti, I. Fiemmeite Cu2(C2O4)(OH)2•2H2O, a new mineral from Val di Fiemme, Trentino, Italy. *Minerals* **8**, 1–10 (2018).

11. Rouse, R. C., Peacor, D. R., Dunn, P. J., Simmons, W. B. & Newbury, D. E. Wheatleyite, Na2Cu(C2O4)2·2H2O, a natural sodium copper salt of oxalic acid. *Am. Mineral.* **71**, 1240–1242 (1986).

12. Chukanov, N. V. *et al.* Antipinite, KNa3Cu2(C2O4)4, a new mineral species from a guano deposit at Pabellón de Pica, Chile. *Mineral. Mag.* **79**, 1111–1121 (2015).

13. Sayer, J. A., Kierans, M. & Gadd, G. M. Solubilisation of some naturally occurring metal-bearing minerals, limescale and lead phosphate by Aspergillus niger. *FEMS Microbiol. Lett.* **154**, 29–35 (1997).

14. Sokolyanskaya, L. O. *et al.* Copper Precipitation as Insoluble Oxalates by Thermotolerant Aspergillus spp. from Burning Wastes of Coal Mining. *Microbiology* **89**, 498–501 (2020).

15. Murphy, R. J. & Levy, J. F. Production of copper oxalate by some copper tolerant fungi. *Trans. Br. Mycol. Soc.* **81**, 165–168 (1983).

16. Joseph, E. *et al.* Development of an analytical procedure for evaluation of the protective behaviour of innovative fungal patinas on archaeological and artistic metal artefacts. *Anal. Bioanal. Chem.* **399**, 2899–2907 (2011).

17. Joseph, E. *et al.* Protection of metal artifacts with the formation of metal-oxalates complexes by Beauveria bassiana. *Front. Microbiol.* **2**, 1–8 (2012).

18. Joseph, E., Simon, A., Rocco, M., Job, D. & Wörle, M. Spectroscopic characterization of an innovative biological treatment for corroded metal artefacts. *J. Raman Spectrosc.* **43**, 1612–1616 (2012).

19. Fomina, M. *et al.* Role of oxalic acid overexcretion in transformations of toxic metal minerals by Beauveria caledonica. *Appl Env. Microbiol* **71**, 371–381 (2005).

20. Zhao, J., Csetenyi, L. & Gadd, G. M. Biocorrosion of copper metal by Aspergillus niger. *Int. Biodeterior. Biodegrad.* **154:105081**, 10 (2020).

21. Hattori, T. *et al.* Rapid copper transfer and precipitation by wood-rotting fungi can effect copper removal from copper sulfate-treated wood blocks during solid-state fungal treatment. *Int. Biodeterior. Biodegradation* **97**, 195–201 (2015).

22. Fomina, M., Bowen, A. D., Charnock, J. M., Podgorsky, V. S. & Gadd, G. M. Biogeochemical spatio-temporal transformation of copper in Aspergillus niger colonies grown on malachite with different inorganic nitrogen sources. *Environ. Microbiol.* **19**, 1310–1321 (2017).

23. Vlasov, D. Y. *et al.* The use of Aspergillus niger in modeling of modern mineral formation in lithobiotic systems. in *Aspergillus niger. Pathogenicity, cultivation and uses* (ed. Baughan, E.) 1–123 (New York, 2020).

24. Magnuson, J. K. & Lasure, L. L. Organic Acid Production by Filamentous Fungi. in *Advances in Fungal Biotechnology for Industry, Agriculture, and Medicine* 307–340 (Springer, Boston, MA, 2004). doi:https://doi.org/10.1007/978-1-4419-8859-1_12.

25. Sazanova, K., Vlasov, D. Y., Osmolovskay, N., Schiparev, S. & Rusakov, A. Significance and regulation of acids production by rock-inhabited fungi. in *Biogenic—Abiogenic Interactions in Natural and Anthropogenic Systems* (eds. Frank-Kamenetskaya, O., Panova, E. & Vlasov, Dy.) 379–392 (Springer, 2016).

26. Osorio, N. W. & Habte, M. Soil Phosphate Desorption Induced by a Phosphate-Solubilizing Fungus. *Commun. Soil Sci. Plant Anal.* **45**, 451–460 (2014).
